# Role of Cardiac AMP-Activated Protein Kinase in a Non-pathological Setting: Evidence From Cardiomyocyte-Specific, Inducible AMP-Activated Protein Kinase α1α2-Knockout Mice

**DOI:** 10.3389/fcell.2021.731015

**Published:** 2021-10-18

**Authors:** Malgorzata Tokarska-Schlattner, Laurence Kay, Pascale Perret, Raffaella Isola, Stéphane Attia, Frédéric Lamarche, Cindy Tellier, Cécile Cottet-Rousselle, Amjad Uneisi, Isabelle Hininger-Favier, Marc Foretz, Hervé Dubouchaud, Catherine Ghezzi, Christian Zuppinger, Benoit Viollet, Uwe Schlattner

**Affiliations:** ^1^Inserm U1055, Laboratory of Fundamental and Applied Bioenergetics (LBFA), University of Grenoble Alpes, Grenoble, France; ^2^Inserm U1039, Radiopharmaceutiques Biocliniques, Faculté de Médecine, University of Grenoble Alpes, Grenoble, France; ^3^Department of Biomedical Sciences, Division of Cytomorphology, University of Cagliari, Cagliari, Italy; ^4^Institut Cochin, CNRS, INSERM, Université de Paris, Paris, France; ^5^Department of Cardiology, Inselspital, Bern University Hospital, Bern, Switzerland; ^6^Institut Universitaire de France, Paris, France

**Keywords:** AMP-activated protein kinase, AMPK, heart, conditional inducible KO, energetics, exercise, mitochondria

## Abstract

AMP-activated protein kinase (AMPK) is a key regulator of energy homeostasis under conditions of energy stress. Though heart is one of the most energy requiring organs and depends on a perfect match of energy supply with high and fluctuating energy demand to maintain its contractile performance, the role of AMPK in this organ is still not entirely clear, in particular in a non-pathological setting. In this work, we characterized cardiomyocyte-specific, inducible AMPKα1 and α2 knockout mice (KO), where KO was induced at the age of 8 weeks, and assessed their phenotype under physiological conditions. In the heart of KO mice, both AMPKα isoforms were strongly reduced and thus deleted in a large part of cardiomyocytes already 2 weeks after tamoxifen administration, persisting during the entire study period. AMPK KO had no effect on heart function at baseline, but alterations were observed under increased workload induced by dobutamine stress, consistent with lower endurance exercise capacity observed in AMPK KO mice. AMPKα deletion also induced a decrease in basal metabolic rate (oxygen uptake, energy expenditure) together with a trend to lower locomotor activity of AMPK KO mice 12 months after tamoxifen administration. Loss of AMPK resulted in multiple alterations of cardiac mitochondria: reduced respiration with complex I substrates as measured in isolated mitochondria, reduced activity of complexes I and IV, and a shift in mitochondrial cristae morphology from lamellar to mixed lamellar-tubular. A strong tendency to diminished ATP and glycogen level was observed in older animals, 1 year after tamoxifen administration. Our study suggests important roles of cardiac AMPK at increased cardiac workload, potentially limiting exercise performance. This is at least partially due to impaired mitochondrial function and bioenergetics which degrades with age.

## Introduction

Adenosine monophosphate (AMP)-activated protein kinase (AMPK) has been initially described as a key sensor and regulator of energy and nutrient signaling, participating in the maintenance of cellular energy homeostasis (Hardie et al., 1998, 2012; Viollet et al., 2010). The role of AMPK was further extended to the regulation of several other cellular and whole body homeostatic functions that are energy-related (Mihaylova and Shaw, 2011; Hardie et al., 2016; Carling, 2017; Herzig and Shaw, 2018; Lin and Hardie, 2018). These include glucose-sensing at the lysosome, autophagy, mitochondrial biogenesis, as well as cell polarity, excitability, motility, growth, and proliferation (Mihaylova and Shaw, 2011; Hardie et al., 2012; Herzig and Shaw, 2018; Lin and Hardie, 2018).

AMP-activated protein kinase is characterized by an intricate structure and regulation. The heterotrimeric protein complex consists of a catalytic α subunit and two regulatory β and γ subunits. These occur as multiple isoforms (α1, α2, β1, β2, γ1, γ2, and γ3) encoded by distinct genes, with every tissue expressing a specific subset of the 12 possible heterotrimers. In rodent heart, the α2β2γ1 heterotrimer is the most abundant. AMPK activation occurs by collective action of covalent and allosteric mechanisms, which involve upstream kinases (LKB1, predominant in the heart, and CaMKKβ) and phosphatases that act on T172 within the α subunit, as well as increased AMP and ADP levels (resulting from the use of ATP) that favor covalent activation, but also activate via binding to the regulatory γ subunit. Thus, any metabolic stress that impairs ATP production, such as glucose starvation or hypoxia, or increases ATP consumption, such as muscle contraction, will activate AMPK. Once activated, AMPK signaling will compensate energy deficits by stimulating production and decreasing consumption of cellular ATP. Much of this regulation targets metabolic pathways, e.g., by activation of sugar and fatty acid uptake/oxidation, or by inhibition of lipid, carbohydrate and protein synthesis. However, AMPK signaling also acts beyond metabolic pathways via signaling at the organellar and cellular levels.

In the heart, AMPK signaling is supposed to play an important role to support the high energy turnover and remarkable metabolic homeostasis of this organ. However, most studies on cardiac AMPK address pathological conditions, and definite answers for its role in more physiological settings are still scarce [reviewed in Dyck and Lopaschuk (2006); Arad et al. (2007); Zaha and Young (2012); Pelosse et al. (2015); Qi and Young (2015); Salt and Hardie (2017); Marino et al. (2021)]. Available literature suggests that AMPK activation in the heart, in contrast to most other tissues, rather acts as a last safeguard during severe energy deprivation and in pathological situations. Cardiac AMPK is activated by energy stress in pathological situations (ischemia, heart failure, some forms of pressure overload, ethanol or Paraquat toxicity) and during exercise, by intracellular calcium overload, or by reactive oxygen and nitrogen species (though oxidative stress can also inactivate the LKB1/AMPK pathway) (Guo and Ren, 2012; Zaha and Young, 2012; Wang et al., 2014; Marino et al., 2021). Under these conditions, AMPK also regulates cardiac turnover of proteins and organelles, in particular mitochondria (“organellar homeostasis”), critical for the survival and self-renewal and remodeling of terminally differentiated cells like cardiomyocytes (Baskin and Taegtmeyer, 2011; Zaha and Young, 2012) and possibly also linked to cardiac aging (Wang et al., 2019).

New insight into the physiological role of cardiac AMPK has been expected from transgenic mice. Indeed, the development of mouse models of constitutive, germline AMPK deficiency either by kinase knock-out (KO) or overexpression of AMPK kinase-dead mutants (KD) allowed important progress (Viollet et al., 2009). These models identified a protective role of AMPK in ischemia and reperfusion, observed among others with heart and skeletal muscle-specific AMPKα2 KD (Russell et al., 2004), whole body AMPKα2 KO (Zarrinpashneh et al., 2006; Carvajal et al., 2007), and heart and skeletal muscle-specific LKB1 KO (Sakamoto et al., 2006). Few models also revealed an involvement of AMPK in the development of cardiomyopathy [whole body AMPKα2 KO (Zarrinpashneh et al., 2008); muscle-specific AMPKβ1β2 KO (Sung et al., 2015)] and in cardiac oxidative capacity [whole body AMPKα2 KO (Athea et al., 2007)]. Finally, the cardiac disorders caused by natural mutations in the AMPK γ isoforms are further evidence for a central role of AMPK in the heart [reviewed by Salt and Hardie (2017)].

The aim of this work was to obtain more insight into the physiological role of cardiac AMPK by using a new transgenic animal model, the inducible, cardiomyocyte-specific knockout mouse deficient for both α-subunit isoforms of AMPK, α1 and α2. Such a model of complete AMPK ablation has not yet been studied in the heart. Moreover, the inducible KO model circumvents inherent caveats of constitutive knock-outs, such as compensatory mechanisms establishing during embryogenesis and development. Here we studied efficiency and specificity of adult tamoxifen-induced and cardiomyocyte-specific AMPKα1α2 deletion as well as its phenotypic consequences under non-pathological conditions. The AMPK KO mouse showed a large reduction of both α isoforms specific for cardiomyocytes. At baseline and in younger animals, loss of AMPK had no discernable phenotype, including unchanged heart function. However, alterations were observed at higher workload in KO mice under dobutamine treatment, consistent with a lower endurance exercise capacity. At the cellular level, loss of AMPK resulted in reduced oxidative function of cardiac mitochondria, mainly related to complex I, and associated with a shift in mitochondrial cristae morphology from lamellar to mixed lamellar-tubular. With advanced age, 1 year after the loss of AMPK, we observed decreased basal metabolic rate together with a trend to lower locomotor activity and diminished energy reserves.

## Materials and Methods

### Animals

All procedures involving animals were approved by the Animal Ethics Committee of the Université Grenoble Alpes (453#15816, 477#19471) and the French Ministry of Higher Education, Research and Innovation (authorization #15816-2018070218298485, #19471-2019022612196859 v3), conform with related European Community legislation (Directive 2010/63/EU).

**FIGURE 1:**
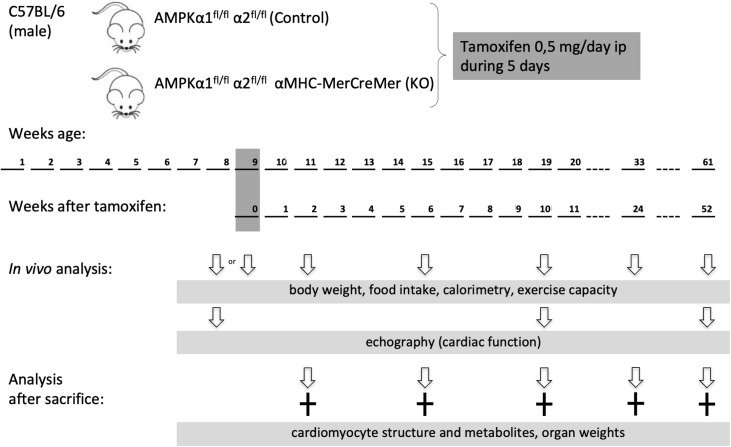
Study design. Eight week old male mice were administered tamoxifen (0.5 mg/day) for five consecutive days and then analyzed at different time points as indicated. Phenotype *in vivo* was followed over 1 year in a group 30 mice (13 control and 17 KO). For other experimental series, additional groups of mice were used, at different time points after KO induction.

AMP-activated protein kinase α1^fl/fl^ α2^fl/fl^ mice were generated as described elsewhere (Boudaba et al., 2018). These mice were crossed with tamoxifen-inducible α-myosin heavy chain (α MHC)–MerCreMer mice (Sohal et al., 2001). At the age of 8 weeks, the offspring [AMPKα1^fl/fl^ AMPKα2^fl/fl^ (αMHC)–MerCreMer mice and AMPKα1^fl/fl^ AMPKα2^fl/fl^] were injected intraperitoneally with tamoxifen (0.5 mg/day) dissolved in corn oil during five consecutive days. Tamoxifen injection induced cardiac specific double KO of AMPK catalytic subunits in AMPKα1^fl/fl^ AMPKα2^fl/fl^ (αMHC)–MerCreMer mice (KO group). Tamoxifen injected AMPKα1^fl/fl^ α2^fl/fl^ littermate mice constituted the control (CTR) group. Male mice were used in the study and analyzed at different time points: 1 week before and 2, 10, 24, or 52 weeks after tamoxifen administration. The experimental scheme is shown in Figure 1.

Mice were maintained under controlled 12 h light/12 h dark cycles (7:00 AM/7:00 PM), 40–70% humidity and a temperature of 24 ± 2°C with free access to water and standard chow (A03, Safe, Augy, France). Mice used for a follow up of the phenotype *in vivo* before and over 1 year after tamoxifen administration were housed in individual cages with environmental enrichment (nesting material, hiding places and toys). For the other experimental series, the usual group housing was chosen.

*In vivo* mice phenotyping included: (i) determination of body weight once a week, at the same time the food intake was evaluated by weighting the feeder of mice kept in individual cages, (ii) echocardiography a week before, and 10 and 52 weeks after tamoxifen administration, (iii) determination of endurance capacity a week before, and 2, 10, and 24 weeks after tamoxifen administration. Fifty-two weeks after tamoxifen administration, this group of mice was sacrificed and hearts used for biochemical analysis.

### Cardiac Echography

Standard procedure: The mouse was anesthetized by isoflurane (Vetflurane, Virbac 1–2% in a 1:1 mixture of O_2_: air). Hair of the thoracic area was removed and the animal was positioned on a heating platform linked to the echography system (Vevo^®^ 2100, VisualSonics) allowing the registration of ECG and respiratory rate. MS-550D (40 MHz; VisualSonics) transducer was used for image acquisition. Standard parameters were obtained allowing the calculation of thickness of cardiac walls, ventricular volumes (during systole and diastole), estimated LV mass, ejection fraction, fractional shortening, cardiac output and stroke volume (Zacchigna et al., 2021). The detailed description of measurements and calculations is provided in Table 1 and Supplementary Table 1.

**TABLE 1:** Cardiac response to dobutamine assessed by echocardiography 10 weeks after induction of KO by tamoxifen.

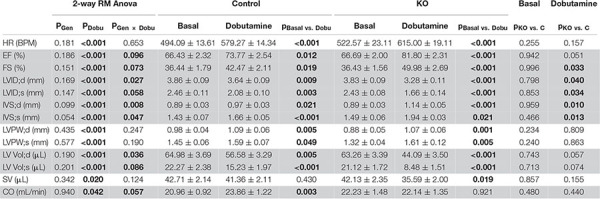

*Data are presented as mean ± SEM (*n* = 11 C and 7 KO) and analyzed by 2-way Repeated Measure ANOVA followed by Holm–Sidak test for pairwise comparisons. Following pairwise comparisons are shown: basal vs. dobutamine within C and KO; and KO vs. C within basal and dobutamine groups. A value of *P* or *p* < 0.05 (for interaction *P* < 0.1) was considered statistically significant (in bold). Highlighted in gray: results with significant interaction (see Materials and Methods).*

***The detailed description of abbreviations, measurements, and calculations:***

*HR, heart rate, ECG.*

***PSLA M-Mode***

*IVS;d, Inter-ventricular septum (diastole), M-Mode.*

*IVS;s, Inter-ventricular septum (systole), M-Mode.*

*LVID;d, Left ventricular internal diameter (diastole), M-Mode.*

*LVID;s, Left ventricular internal diameter (systole), M-Mode.*

*LVPW;d, Left ventricular posterior wall (diastole), M-Mode.*

*LVPW;s, Left ventricular posterior wall (systole), M-Mode.*

***Calculation***

*EF, LV ejection fraction, 100 × ((LV Vol;d-LV Vol;s)/LV Vol;d).*

*FS, LV fractional shortening, 100 × ((LVID;d-LVID;s)/LVID;d).*

*LV Vol;d, Left Ventricle volume diastole, (7.0/(2.4 + LVID;d)) × LVID;d^3^.*

*LV Vol;s, Left Ventricle volume systole, (7.0/(2.4 + LVID;s)) × LVID;s^3^.*

*SV, Stroke volume, LV Vol;d – LV Vol;s.*

*CO, Cardiac output, (SV × HR)/1000.*

Pharmacological stress protocol: The basal measurements were realized after stabilization of the heart rate (HR), around 10 min after anesthesia induction. When finished, an intraperitoneal injection of Dobutamine^®^ was performed (Panpharma, diluted in saline; 0.75 μg/g) (Puhl et al., 2016). Echocardiographic B- and M-Mode scans in long-axis projections and ECG recordings were repeated immediately after the single bolus injection and then periodically during 10 min, until the peak heart rate response was reached and heart rate began to decline. The response to the dobutamine stimulation was slightly different from one mouse to another, in terms of amplitude and timing. If the delta HR was found to be inferior to 30 BPM, the mouse was not included in the final results.

### Tissue Harvesting for Biochemical Analysis

After anesthesia by intraperitoneal injection of ketamine/xylazine (130/10 mg/kg) and tracheotomy, the animal was placed under respirator-supported ventilation and hearts were freeze-clamped *in situ* immediately after thoracotomy using a Wollenberger clamp. Frozen hearts were stored at −80°C.

### Metabolite Determination

For adenine nucleotides and PCr determination, protein-free extracts were obtained by perchloric acid precipitation and metabolite quantification was performed by HPLC (ATP, ADP, and AMP) and spectrophotometric assay (PCr) as described earlier (Tokarska-Schlattner et al., 2010; Gratia et al., 2012). To determine glycogen, heart tissue was digested with KOH, glycogen was precipitated with ethanol and degraded to glucose by acid hydrolysis. Resulting glucose was determined enzymatically using hexokinase and G6PDH in spectrophotometric test. Metabolite content was normalized by wet weight of tissue.

### Oxidative Stress Markers

Markers of oxidative damage were quantified in heart extracts prepared as described earlier (Hininger-Favier et al., 2009). Reduced thiol (SH) groups were assayed as published (Faure and Lafond, 1995). N-acetyl cysteine (NAC) in the range of 0.125–1 mM (prepared from a 100 mM stock solution) was used for calibration. Standards and heart extracts were diluted in 50 mM phosphate buffer, 1 mM EDTA, pH 8 and 2.5 mM 2-acid nitrobenzoic 5,5’-dithio-bis (2-nitrobenzoic acid) (DTNB), and subsequently the absorbance was measured at 412 nm. Plasma thiobarbituric acid reactive substances (TBARS) concentrations were assessed as described (Richard et al., 1992).

### Cardiomyocyte Isolation and Size Determination

Mouse cardiomyocytes were isolated using a standard protocol based on enzymatic procedures (Mitra and Morad, 1985; Wolska and Solaro, 1996) with minor modifications. After isolation, the cardiomyocytes were placed in a Petri dish and the images were captured with an inverted brightfield microscope (Axiovert). The images were used to analyze cardiomyocyte size by ImageJ software.

### Electron Microscopy

Electron microscopy was performed as described earlier (Isola et al., 2013; Loy et al., 2014). Briefly, after animal euthanasia and heart excision, pieces of left ventricle of about 1–2 mm size were fixed in 1% paraformaldehyde and 1.25% glutaraldehyde for 2 h. Then, they were stained overnight with 0.75% uranyl acetate, dehydrated in a graded acetone series and embedded in epoxy resin. Specimens were cut with an ultramicrotome at a thickness of 90 nm and counterstained with uranyl acetate and lead citrate. Observation and acquisition of micrographs were performed in a S100 Jeol Transmission Electron Microscope.

### Isolation and Analysis of Mitochondria

Mitochondria were isolated by differential centrifugation. The whole procedure was performed at 4°C. After cervical dislocation hearts were quickly excised, atria were discarded and ventricles were finely chopped in 5 mL of ice-cold isolation buffer (150 mM sucrose, 75 mM KCl, 50 mM Tris Base, 1 mM KH_2_PO_4_, 5 mM MgCl_2_, 1 mM EGTA, pH 7.4) supplemented with 0.2% bovine serum albumin (BSA). The suspension was then incubated with 1 mg/5 mL subtilisin for 1 min and then homogenized using a Potter-Elvehjem glass–Teflon homogenizer at 300 rpm. Homogenate was diluted with 7 mL of isolation buffer to stop the action of subtilisin, and then centrifuged at 800 × *g*, 4°C for 10 min, to remove nuclei and cellular debris. Supernatant was filtered and then centrifuged twice at 8000 × *g*, 4°C for 10 min. Resulting mitochondrial pellet was resuspended in 0.1–0.2 mL isolation buffer. Mitochondrial protein content was determined using Pierce BCA protein assay (Thermo Scientific) with BSA as a standard.

Mitochondrial oxygen consumption was measured at 30°C with a Clark-type electrode (Oxygraph, Hansatech Instruments Ltd., Norfolk, United Kingdom) in a respiration buffer containing 125 mM KCl, 20 mM Tris Base, 1 mM EGTA, pH 7.2 supplemented with 0.2% BSA and 10 mM Pi. Mitochondria were present at 0.2 mg/mL. Resting respiration (State 2) was induced either by 5 mM glutamate/2.5 mM malate (substrates of complex I) or 5 mM succinate (substrates of complex II, in presence of 4 μM rotenone) or 60 μM palmitoylcarnitine (lipid substrate, in presence of 1 mM malate). Subsequently, sequential addition of 1 mM ADP and 0.5 mg/mL oligomycin or 100 μM DNP let assess respectively state 3, state 4 or uncoupled respiration.

To determine activity of cytochrome C oxidase, mitochondria in respiration buffer as above were supplemented sequentially with 1 mM antimycin A, 2.5 mM ascorbate, 25 mM TMPD/10 mM ascorbate and 100 μM DNP.

Activity of complex I was determined spectrophotometrically using frozen mitochondria (thawed and diluted to 8 μg per 20 μL) added to assay buffer consisting of 50 mM K_2_HPO_4_, 1 mM EDTA, 0.4% BSA, 0.1 mM NADH, 100 μM decylubiquinone. Reaction was followed at 37°C, 340 nm for 3 min; baseline was measured after inhibition of complex I with 10 μM rotenone.

Total carnitine palmitoyltransferase (CPT1 + CPT2) activity was measured according to Boudina et al. (2005) using 200 μg of isolated mitochondria in 1 mL of assay buffer containing 20 mM HEPES, 1 mM EGTA, 220 mM sucrose, 40 mM KCl, 0.1 mM DTNB, 1.3 mg/mL BSA, and 40 μM palmitoyl-CoA, pH 7.4 at 37°C. The reaction was started by adding 1 mM carnitine and was monitored at 412 nm for 4 min.

### Calcium Retention Assay

Ca^2+^ measurements were performed fluorimetrically with a PTI Quantamaster C61 spectrofluorometer equipped with magnetic stirring and thermostatic controls. Extra-mitochondrial Ca^2+^ was measured at 30°C in the presence of 0.25 μM Calcium Green-5N with excitation and emission wavelengths set at 506 and 530 nm, respectively. Mitochondrial Ca^2+^ uptake and Ca^2+^ release were measured by loading mitochondria with trains of Ca^2+^ pulses at constant time intervals (Fontaine et al., 1998).

### Immunoblotting

Sodium dodecyl sulphate (SDS)-polyacrylamide gel electrophoresis (PAGE) separation of heart extracts and immunoblotting was performed according to the standard procedures. Briefly, the frozen tissue was homogenized in ice-cold lysis buffer (Mammalian protein extraction buffer, GE Healthcare) complemented with 1 mM EDTA, 1 mM EGTA, and 1 mM DTT, protease inhibitor cocktail (Roche) and Halt phosphatase inhibitor cocktail (Thermo Scientific) using bead cell disruptor (Retsch-MM301). The homogenate was placed on ice for 20 min and then centrifuged at 14,000 × *g* for 20 min at 4°C. The resulting supernatant was recovered and stored at −80°C. The protein concentration was determined by Bradford method using Bio-Rad reagent with BSA as a standard. 40 μg of proteins were denaturated using an SDS-PAGE-DTT loading buffer, heated at 95°C for 5 min and separated on a polyacrylamide gel (Mini-protean TGX 4–15%, Bio-Rad) at 200 V for 45–55 min. Then, the proteins were transferred onto a nitrocellulose membrane by a semi-dry transfer system (*Trans*-Blot Turbo, Bio-Rad) at 25 V, 1.3 A for 10 min. After transfer, its quality and equal loading were checked by the Ponceau staining. After destaining, the membrane was saturated with 4% skim milk in TBS-Tween 0.1% for 2 h at room temperature (RT) and incubated with a primary antibody (2 h at RT or overnight at 4°C). Then the membrane was washed with TBS-Tween 0.1% and incubated with an HRP-conjugated secondary antibody for 1 h at RT. The blots were developed with chemiluminescence reagent (ECL Prime, GE Healthcare) using CCD camera (ImageQuant LAS 4000, GE Healthcare). The quantification of signals was done using ImageQuant TL software (GE Healthcare). Tubulin, actin or total protein for the phosphorylated proteins (total and phosphorylated protein were probed on different membranes) signals were used for normalization.

Following primary antibodies were used: anti-AMPKα1, anti-AMPKα, anti-P-AMPKα (T172), anti-ACC, anti-P-ACC (Ser79), anti-tubulin and anti-actin from Cell Signaling Technology. Anti-AMPKα2 was from MRC PPU Reagents and Services (Dundee, United Kingdom), anti-AMPKβ2 and anti-AMPKα1β1γ1 (used to probe γ1) were a kind gift of Prof. Graham Hardie (University of Dundee, United Kingdom).

### Immunofluorescence in Heart Cryosections

After anesthesia by intraperitoneal injection of ketamine/xylazine (130/10 mg/kg) and heparin (1500 UI/kg), hearts were perfused *in situ* with PBS during 1 min and harvested. The left ventricle tissue was fixed with 4% PFA in PBS during 2 h at RT, infiltrated with 10–30% sucrose for cryoprotection, embedded in OCT-Tissue Freezing Medium (MM, France) and frozen in liquid nitrogen. Sections of 5–10 μm were cut with cryostat and mounted on histological slides (Superfrost Plus Gold, Thermo Scientific). Cryosections were washed with PBS, fixed in 4% PFA for 5 min at RT, permeabilized in 0.2% Triton-PBS during 5 min and washed again with PBS. After blocking in BSA/PBS (1 mg/mL) for 15 min at RT, sections were incubated with primary antibodies diluted in BSA/PBS (1 mg/mL) overnight at 4°C. After washing with PBS for 3 × 5 min, sections were incubated with secondary antibodies diluted in BSA/PBS (1 mg/mL) for 1 h at RT in obscurity. The sections were further washed in PBS and mounted with Vectashield medium containing DAPI for nuclei labeling (Eurobio, France). Acquisition of the images was carried out with a Leica TCS SP2 AOBS confocal microscope (LEICA Microsystems Heidelberg, Germany) equipped with a HCX PL APO CS 63×/1.40 oil objective and driven by LasX software. Laser excitations were 350–364 nm, 488 nm, 543 nm and 638 nm and fluorescence emissions were precisely collected between 420 and 470 nm, 500 and 540 nm, 560 and 660 nm, and 650 and 690 nm for DAPI, FITC or Dylight488, rhodamine and Cy5, respectively. Optical sections were acquired by the mean of 9–12 μm z-stacks with a z-step of 1 μm and confocal pinhole size was at 1 (Airy units) for all channels.

Following primary antibodies were used (all at 1:100 dilution): rabbit anti-AMPKα1 (Cell Signaling Technology), sheep anti-AMPKα2 (MRC PPU Reagents and Services, Dundee, United Kingdom), mouse anti α-smooth muscle actin and mouse anti sarcomeric α-actinin (Abcam), rabbit anti-laminin (Sigma), rabbit anti N-cadherin (Santa Cruz Biotechnology). Secondary antibodies (used at 1:200 dilution) were from Jackson: anti-mouse coupled to rhodamine or to Cy5, anti-rabbit coupled to FITC or to Dylight488, anti-sheep coupled to rhodamine.

### Endurance Capacity Testing

For endurance capacity testing, mice were familiarized with a motorized treadmill (Bioseb) by running for 5 min at 15 m/min (without incline) 1 day preceding the test. On day of testing, mice run until exhaustion on treadmill according to the following protocol: 5 min at 15 m/min, 30 min at 18 m/min and then 20 m/min, all without incline. Mice were motivated to run by light electrical stimuli (0.1–0.6 mA) according to the manufacturer’s recommendations. During exercise mice were continuously monitored by the experimented technician (visible fatigue associated with a change in running style, tail and hind haunches lowered, inability to re-start running after more than 3 aversive stimuli within 10 s). The performance corresponded to the running time-to-exhaustion and was expressed in min.

### Moderate Running Exercise

In experiments aimed to evaluate impact of exercise on cardiac energy status, mice performed a running exercise at a moderate intensity (fixed speed 18 m/min without incline) for a fixed time of 30 min. During the week preceding exercise, mice were familiarized with the treadmill (Bioseb) by running every day for 5 min at 15 m/min without incline; animals were motivated to run by light electrical stimuli (0.6 mA). During exercise mice were continuously monitored by the experimented technician (see above). At the end of exercise, mice were put back into their cages for 5 min to recover (water access *ad libitum*) and then heart and skeletal muscles were harvested as described above. Mice, which were not able to complete exercise, were excluded from the analysis.

### Calorimetry

Energy expenditure was determined using indirect calorimetry. Oxygen consumption (VO_2_), carbon dioxide production (VCO_2_) and respiratory exchange ratio (RER) were monitored using an indirect open circuit calorimeter in individual cages over 36 h (LE 405, Panlab-Bioseb). Airflow rate was obtained from a gas pump and a flow meter. Data were recorded using a computer-assisted data acquisition program (Metabolism Calculation Software, Version 2.0.2). Energy expenditure (EE) was calculated according to the following formula provided by the supplier: EE = (3.815 + 1.232 × VO_2_/VCO_2_) × VO_2_ × 1.44 and was expressed in kcal/kg/h of body weight.

### Spontaneous Locomotor Activity

Spontaneous locomotor activity of mice was measured by video-tracking of the animals in their usual cages as described earlier (Chabert et al., 2016).

### Statistics

Results are expressed as means ± SEM, if not stated otherwise. To compare two groups, Student’s *t*-test was performed if requirements of normal distribution and equality of variance were fulfilled; if not, Mann–Whitney test was used. Other statistical analyses were performed with two-way ANOVA (or repeated measure two-way ANOVA, when measurements were taken on the same individuals at different time points/conditions) to see if experimental groups are affected by two different factors. In our experiments, one factor was systematically genotype, the second factor was variable depending on experiment (time, light/dark period, exercise, dobutamine). For two-way ANOVA, we report significance values for two main effects of the two factors (P_*Factor*__1_, P_*Factor*__2_; main effects deal with each factor separately, i.e., describe comparisons within the levels each factor, when ignoring the levels of another factor), interaction of the two factors (P_*Factor*1 × *Factor*2_; indicating if the effect of one factor depends on the level of the second factor), and Holm–Sidak or Student–Newman–Keuls test for pairwise comparisons (p). Statistics was performed using Sigma Plot (Systat Software, San Jose, CA, United States). In figures, *P*- and *p*-values are given in gray with two digits after comma when not significant, and in black with three digits after comma when significant. A value of *P* or *p* < 0.05 (for interaction *P* < 0.1) was considered statistically significant.

## Results

### Efficiency and Specificity of Adult Inducible and Cardiomyocyte-Specific of AMP-Activated Protein Kinase α Deletion

**FIGURE 2:**
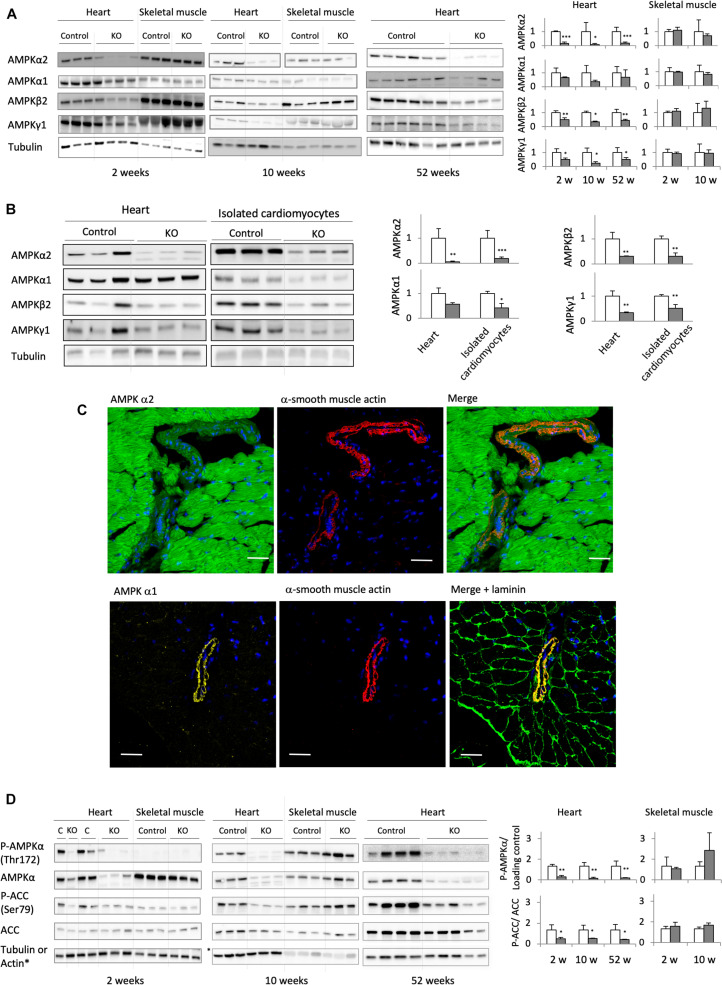
Cardiomyocyte-specific AMPKα1 and α2 deletion. **(A)** Immunoblot analysis of the expression of AMPK subunits (α1, α2, β2, and γ1) in total heart extract of control and AMPKα1α2-KO mice 2, 10, and 52 weeks after tamoxifen administration; for comparison, the expression of AMPK subunits in skeletal muscles at 2 and 10 weeks is given. **(B)** Western blot detection of AMPK subunits in total heart and isolated cardiomyocytes from control and AMPKα1α2-KO mice 6 weeks after tamoxifen administration. For each protein, signals for total heart and isolated cardiomyocytes originate from the same blot. **(C)** Distribution of AMPKα1 and α2 isoforms between cardiomyocytes and smooth muscle cells of blood vessels (stained with α-smooth muscle actin) studied by immunofluorescence in control heart sections. Laminin staining was used to visualize cell borders. Scale bar 30 μm. As seen here, remnants of AMPKα2 and mainly α1 in AMPKα1α2-KO total heart extract in panel **(A)** are likely due to the presence of non-cardiomyocyte cells in heart tissue. **(D)** Phosphorylation of AMPKα (Thr172) and ACC (Ser79) in total heart extracts of control and AMPKα1α2-KO mice 2, 10, and 52 weeks after tamoxifen administration; for comparison, P-AMPKα and P-ACC in skeletal muscles at 2 and 10 weeks are given. For panels **(A,B,D)**: representative tubulin blots for each sample are shown, further loading controls are given in Supplementary Figure 2; band quantifications were corrected for loading (tubulin, actin or total protein); control (white bar, normalized to 1), KO (gray bar). Data are presented as means ± SD. ^∗^*p* < 0.05, ^∗∗^*p* < 0.01, ^∗∗∗^*p* < 0.001 vs. control (*n* ≥ 3) as determined by *t*-test.

Deletion of the catalytic α1 and α2 subunits of AMPK (double knock-out, KO) was induced by injecting tamoxifen to adult, 8 weeks old mice carrying floxed α subunits and cardiomyocyte-specific expression of Cre (AMPKα1^*fl/fl*^ AMPKα2^*fl/fl*^ (α-MHC)–MerCreMer). Tamoxifen-treated mice without Cre (AMPKα^*fl/fl*^ AMPKα2^*fl/fl*^) were used as controls (CTR). While efficient AMPK deletion took place in AMPKα1^*fl/fl*^ AMPKα2^*fl/fl*^ (α-MHC)–MerCreMer mice after tamoxifen administration, injection of vehicle to these animals had no effect, confirming that the inducible Cre system was not leaky (Supplementary Figure 1). Cardiac expression of α1 and α2 subunits was verified at all sampling intervals used in this study, i.e., 2, 6, 10, 24, and 52 weeks after tamoxifen administration (Figures 2A,B; data for 24 weeks not shown). As shown in immunoblots of total heart extracts, both α1 and α2 subunits were largely deleted in KO mice already after 2 weeks, persisting until 52 weeks. The major cardiac AMPKα isoform, α2, was decreased by about 80–90% as compared to CTR, while the effect on α1 was less pronounced. This is largely due to non-cardiomyocyte cells present in cardiac tissue like fibroblasts or endothelial cells that express predominantly the α1 isoform. Immunofluorescence analysis of cardiac thin sections (Figure 2C) clearly show the predominant expression of α2 in cardiomyocytes as compared to non-cardiomyocyte cells, while endothelial and smooth muscle cells surrounding blood vessels express mainly α1 (Figure 2C). We therefore isolated cardiomyocytes from KO and CTR animals (Figure 2B). Immunoblot analysis confirmed an enrichment of AMPKα2 in cardiomyocytes as compared to total heart, and a large decrease of both AMPKα2 and α1 isoforms after KO induction (Figure 2B). In the KO heart, basal activation of AMPK as analyzed by αThr172 phosphorylation in immunoblots almost disappeared, and phosphorylation of the reference AMPK substrate ACC at Ser79 was largely diminished (Figure 2D). Immunoblots further revealed a concomitant decrease of AMPK β and γ subunits, e.g., AMPK β2 and AMPK γ1 (Figures 2A,B). Likely, in absence of α subunits, β and γ cannot fold and/or form stable subcomplexes and are degraded. Collectively, these data suggest that the largest portion of cardiomyocytes has lost expression of the entire AMPK heterotrimeric complex. This loss of AMPK (Figure 2A) and AMPK or ACC phosphorylation (Figure 2D) was specific to the heart, since no changes were observed in skeletal muscles.

### Body Weight, Food Intake, Locomotor Activity, and Metabolic Rate

**FIGURE 3:**
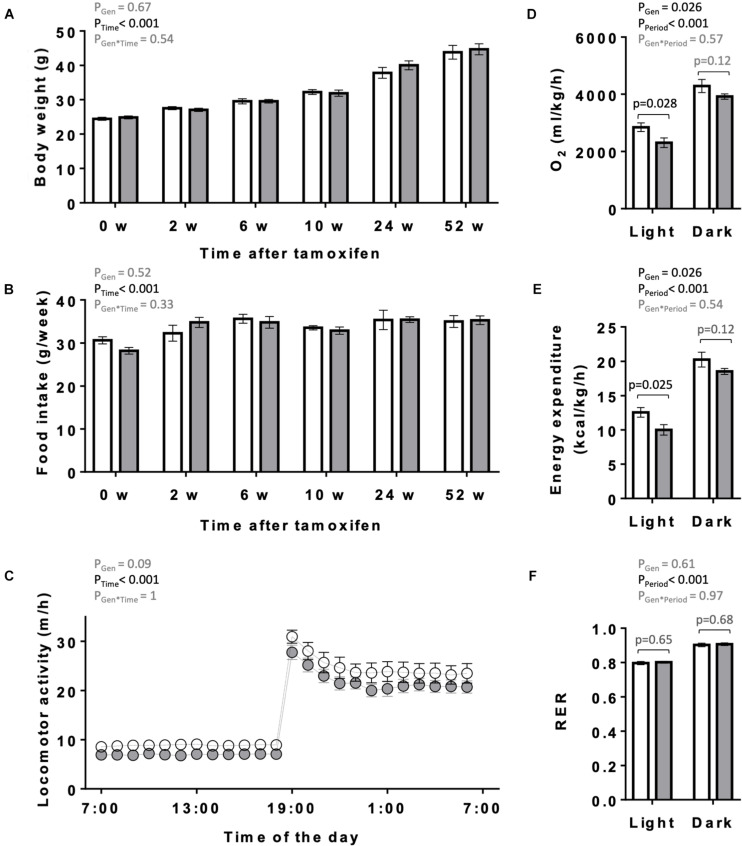
Body weight, food intake, locomotor and metabolic activity in control and AMPKα1α2-KO mice. **(A,B)** Body weight **(A)** and food intake **(B)** of mice of the both genotypes on the day of the first tamoxifen injection and then 2, 6, 10, 24, and 52 weeks after tamoxifen administration. **(C)** Diurnal locomotor activity of control and AMPKα1α2-KO mice 12 months after tamoxifen administration. **(D–F)** Metabolic activity of control and AMPKα1α2-KO mice 12 months after tamoxifen administration: oxygen consumption **(D)**, energy expenditure **(E)** and respiratory exchange ratio (RER, **F**). Control (white bar and symbols), KO (gray bar and symbols). Data are given as means ± SEM; *n* = 13 control and 17 KO **(A,B)**, 5 control and 9 KO **(C–F)**. Data were analyzed by 2-way Repeated Measure ANOVA **(A–F)** followed in panel **(D–F)** by Student–Newman–Keuls test for pairwise comparisons. Resulting significance values are given for two main effects related either to genotype P_*Gen*_, time P_*Time*_, and their interaction P_*Gen* × *Time*_ **(A–C)** or to genotype P_*Gen*_, light/dark period P_*Period*_, and their interaction P_*Gen* × *Period*_ **(D–F)**; in panel **(D–F)** significance values, *p*, of pairwise comparisons between two genotypes are given for light and dark periods. For more details see section “Materials and Methods.”

The overall appearance of the KO mice was normal. Body weight and food intake of KO and CTR mice were analyzed over 1 year following tamoxifen injection (Figures 3A,B). They remained similar between both genotypes over the entire study period, although a non-significant trend to higher body weight may be present in older KO animals (Figure 3A). One year after tamoxifen administration, spontaneous locomotor activity showed a tendency to decrease in KO mice (two-way ANOVA significance P_*G*__*en*_ = 0.09; Figure 3C). We further determined in these mice the basal, average diurnal metabolic rate by measuring oxygen consumption and energy expenditure. These were lower in KO than in CTR mice, reaching significance in the light period (Figures 3D,E). The RER remained unchanged between KO and CTR animals (Figure 3F).

### Cardiac Function at Baseline and After Dobutamine Stimulation

**FIGURE 4:**
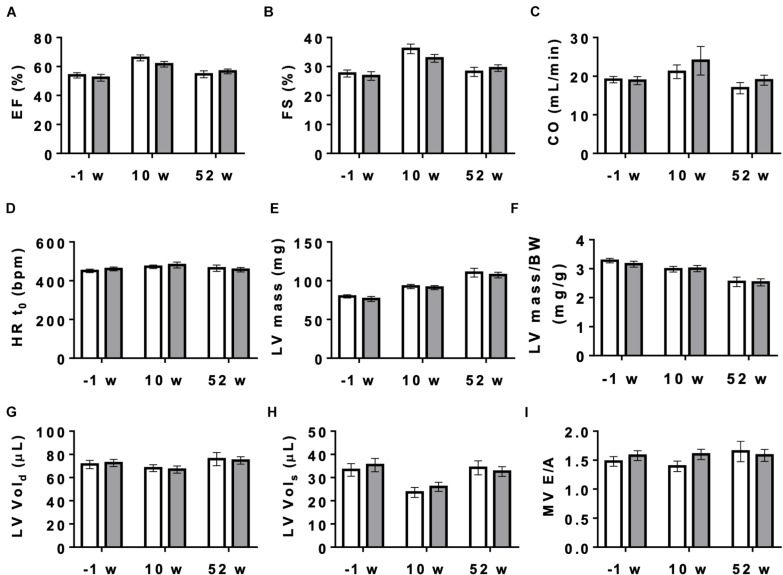
Cardiac function measured by echocardiography in control and AMPKα1α2-KO mice. Cardiac function in mice of both genotypes measured 1 week before tamoxifen administration and then 10 and 52 weeks after last tamoxifen injection: **(A)** EF, ejection fraction, **(B)** FS, fractional shortening, **(C)** CO, cardiac output, **(D)** HRt_0_, heart rate t_0_, **(E)** LV mass corrected, **(F)** LV mass corrected/BW, **(G)** LV Vol;d, left ventricle volume diastole, **(H)** LV Vol;s, left ventricle volume systole, **(I)** MV E/A, mitral valve E/A ratio. Control (white bar), KO (gray bar). Data, given as means ± SEM (*n* = 11 CTR, 16 KO) were analyzed by 2-way Repeated Measure ANOVA, followed by Holm–Sidak test for multiple comparisons. For all parameters shown, the effects of genotype and interaction between genotype and time were not significant, the effect of time is significant (*P* < 0.001) for EF, FS, LV mass, LV mass/BW and LV Vol_*s*_. The entire set of functional data, results of statistical analysis and detailed description of measurement and calculations are provided in Supplementary Table 1.

Next, we studied the effect of AMPK deletion on cardiac function in anesthetized CTR and KO mice using transthoracic echography. At baseline conditions, 10 and 52 weeks after tamoxifen administration, many of the examined functional parameters showed significant age-related changes, but there were no significant differences in cardiac function between CTR and KO mice (Figure 4 and Supplementary Table 1). We then studied cardiac function at increased workload, using dobutamine injection as an acute β-adrenergic stimulation model. This is a powerful tool to characterize the cardiovascular phenotype of transgenic mice and to reveal cardiac dysfunction in animals that appear normal under resting conditions (Wiesmann et al., 2001). Indeed, dobutamine stimulation revealed certain deleterious consequences of AMPK deletion (Table 1). The response to dobutamine was significantly altered in KO mice, as seen for different functional parameters (Table 1). In particular, in contrast to controls, KO mice were unable to increase cardiac output in response to dobutamine-induced workload.

### Exercise Capacity

**FIGURE 5:**
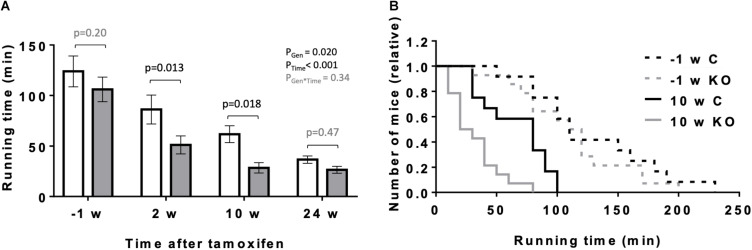
Effect of cardiomyocyte-specific AMPKα1 and α2 deletion on exercise capacity of mice. **(A)** Exercise capacity (running time till exhaustion) of mice of the both genotypes 1 week before and 2, 10, and 24 weeks after tamoxifen administration. Control (white bar), KO (gray bar). **(B)** Frequency distributions of till exhaustion running times in mice of the both genotypes 1 week before and 10 weeks after tamoxifen administration. Data are presented as means ± SEM; *n* = 12 control and 14 KO. In panel **(A)** data were analyzed by 2-way Repeated Measure ANOVA followed by Holm–Sidak test for pairwise comparisons. Resulting significance values are given for two main effects related to genotype P_*Gen*_, time P_*Time*_, and their interaction P_*Gen* × *Time*_; pairwise comparisons between two genotypes are performed for different time points; the resulting *p*-values are given.

In the following, we analyzed the performance of KO and CTR mice under a more physiologically increased workload like exercise. We compared the maximal exercise capacity of mice before and at 2, 10, and 24 weeks after tamoxifen administration using a treadmill setup (Figure 5). Endurance exercise capacity was determined by the ability of mice to perform prolonged treadmill running, measured as maximal running time. As anticipated, maximal running time decreased significantly with the age of mice (Figure 5A). However, the decrease in maximal running time was more pronounced for KO animals as compared to CTR, and significant differences were measurable at 2 and 10 weeks after KO induction (Figures 5A,B).

### Cardiomyocyte Size and Cytoarchitecture

**FIGURE 6:**
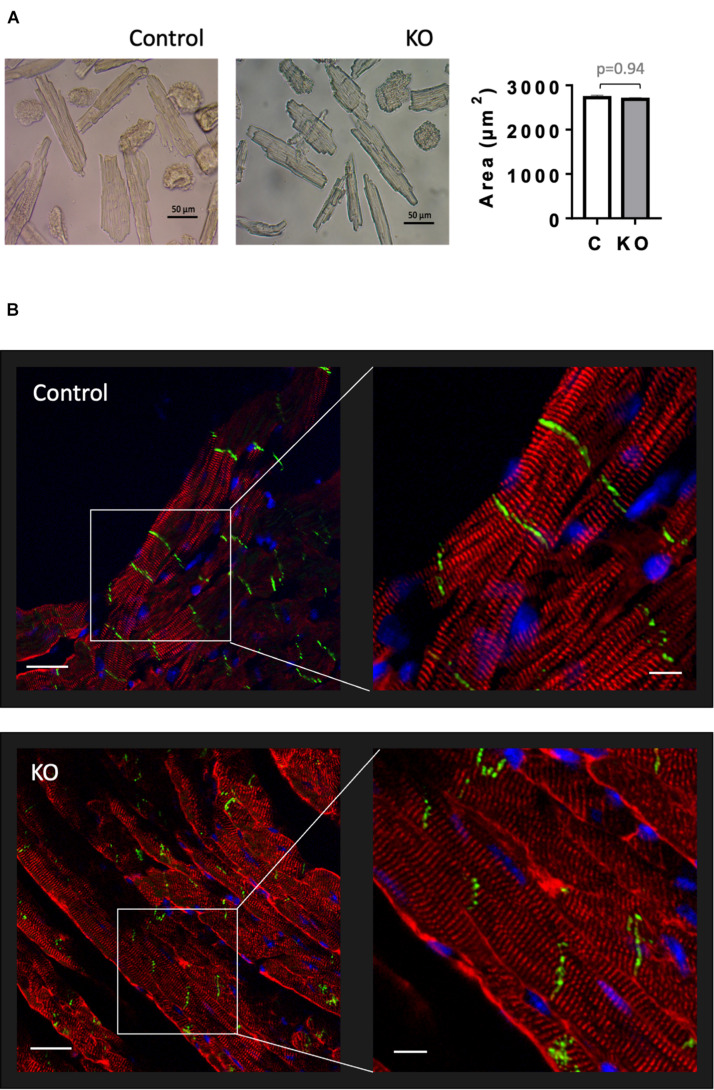
Cardiomyocyte size and cardiac cytoarchitecture in control and AMPKα1α2-KO mice. **(A)** Isolated cardiomyocytes from control and AMPKα1α2-KO mice 6 weeks after tamoxifen administration; the images were captured by an inverted brightfield microscope and quantified using ImageJ. Scale bars are 50 μm. Control (white bar), KO (gray bar). The size of isolated cardiomyocytes is given as mean ± SEM (*n* = 3 hearts from each group; 100 cardiomyocytes/heart); comparison between two groups was done using *t*-test. **(B)** Immunofluorescence confocal microscopy of heart cryosections stained with the Z-disk protein sarcomeric α-actinin (red), the cell-cell contact protein N-cadherin (green) and the nuclear stain DAPI (blue). Scale bars are 30 and 10 μm for left and right panels, respectively.

Given that AMPK signaling was suggested to control cardiac hypertrophy (Salt and Hardie, 2017), we analyzed cardiomyocyte morphology in CTR and KO mice (Figure 6). However, there was no difference in cardiomyocyte size (Figure 6A) and cytoarchitecture (Figure 6B). Consistent with these morphological observations, estimation of left ventricular mass by echocardiography confirmed the lack of cardiac hypertrophy in KO mice (Figure 4E and Supplementary Table 1). The weight of other analyzed organs was also unchanged (Supplementary Table 2).

### Cardiac Energy Metabolites

**FIGURE 7:**
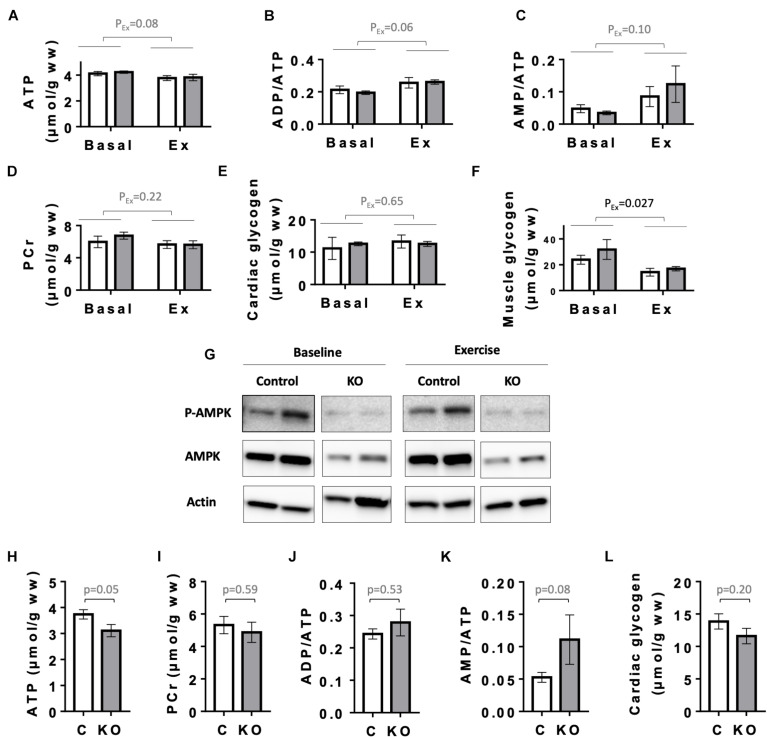
Energy status of control and AMPKα1α2-KO mice. **(A–E)** Basal and post-exercise levels of cardiac energy metabolites 24 weeks after tamoxifen administration: ATP **(A)**, adenine nucleotide ratios ADP/ATP **(B)**, AMP/ATP **(C)**, phosphocreatine **(D)**, and glycogen **(E)**. **(F)** Basal and post-exercise glycogen in skeletal muscle. In panels **(A–F)** all mice were subjected to the same running protocol, i.e., running of moderate intensity at 18 m/min without incline for 30 min. **(G)** Phosphorylation of AMPKα (Thr172) in hearts analyzed in panels **(A–F)**; for each antibody, all signals originate from the same blot. **(H–L)** Cardiac energy metabolites 52 weeks after tamoxifen administration: ATP **(H)**, phosphocreatine **(I)**, adenine nucleotide ratios ADP/ATP **(J)**, AMP/ATP **(K)**, and cardiac glycogen **(L)**. Control (white bar), KO (gray bar). Data are presented as means ± SEM. In panels **(A–F)** *n* = 4 control and 3 KO (baseline), 5 control and 4 KO (exercise). Data were analyzed by 2-way ANOVA and the resulting significance values are given only for the main effects of exercise P_*E*__*x*_; the effects of genotype and interaction between genotype and exercise are not significant for all parameters in panels **(A–F)** and are not reported. In panels **(H–L)**, *n* = 9 **(H–K)**, 13–17 **(L)** and comparisons were performed either using *t*-test or in panels **(J,K)** Mann–Whitney test; the resulting *p*-values are given.

Next, as a primordial physiological parameter for the heart, we examined cardiac energy status. We first analyzed the levels of high energy phosphates ATP and phosphocreatine (PCr), the relevant adenine nucleotides ratios (ADP/ATP and AMP/ATP), as well as glycogen levels in the hearts of CTR and KO mice at baseline and after exercise (Figure 7). Since CTR and KO markedly differed in their running capacity, we chose to compare energy metabolites after a moderate intensity running for a fixed time, which could be fully performed by both genotypes. In this setup, 6 months after tamoxifen administration, there were no significant genotype-related differences in cardiac adenine nucleotides, PCr and glycogen, already at baseline, and also after exercise (Figures 7A–E). Effects of exercise, e.g., decreased ATP or increased ADP/ATP and AMP/ATP ratios, were at the limit of significance (*p* = 0.08, *p* = 0.06, and *p* = 0.10, respectively). Indeed, the moderate intensity running did not impose an energy stress that would activate AMPK even in CTR hearts, as shown by immunoblots of AMPKαThr172 phosphorylation (Figure 7G). We therefore verified whether the applied exercise protocol induced the well-established decrease in skeletal muscle glycogen (Hearris et al., 2018; Vigh-Larsen et al., 2021). Indeed, our exercise protocol decreased glycogen in gastrocnemius muscle of both genotypes (Figure 7F). Interestingly, 1 year after tamoxifen administration, we found a strong tendency to diminished cardiac energy reserves in KO as compared to CTR mice (Figures 7H–L). This included a decrease in baseline ATP (*p* = 0.05), glycogen (*p* = 0.20) and an increased AMP/ATP ratio (*p* = 0.08), indicating a slow onset of detectable energy defects with age in KO mice.

### Mitochondrial Respiratory Function and Oxidative Stress Markers

**FIGURE 8:**
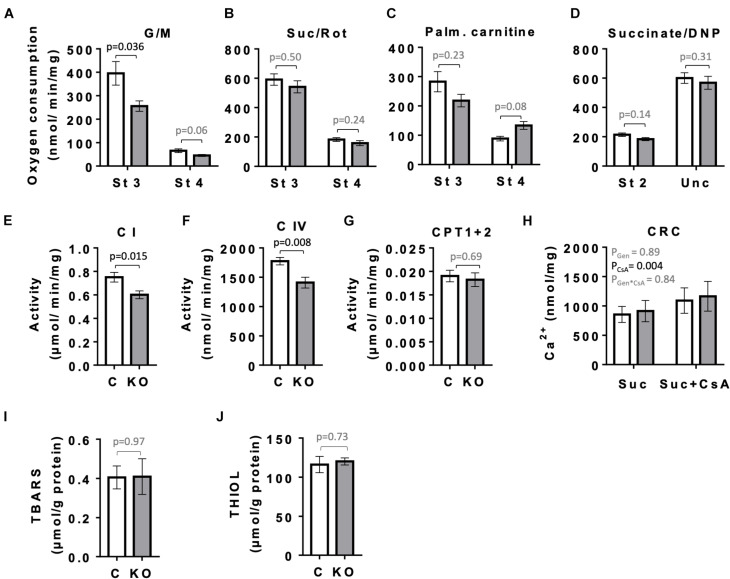
Mitochondrial function and markers of oxidative stress in control and AMPKα1α2-KO mice. **(A–H)** Function of cardiac mitochondria isolated 10 weeks after tamoxifen administration. State 3 and 4 respiration with glutamate and malate **(A)**, succinate in presence of rotenone **(B)**, palmitoylcarnitine **(C)** and DNP-uncoupled respiration with succinate as substrate **(D)**. Activity of mitochondrial enzymes: complex I **(E)**, complex IV **(F)**, carnitine palmitoyltransferase **(G)**. Calcium retention capacity (CRC) in presence and absence of cyclosporine A, CsA **(H)**. **(I,J)** Markers of oxidative stress 52 weeks after tamoxifen administration: TBARS **(I)** and THIOLS **(J)**. Control (white bar), KO (gray bar). Data are presented as means ± SEM; *n* = 5–13 **(A–G)**, 4–5 **(H)**, 10–13 **(I)**, 5 **(J)**. For **(H)** statistical analysis was done by 2-way Repeated Measure ANOVA; the resulting significance values are given for two main effects of genotype P_*Gen*_ and presence of cyclosporine A P_*CsA*_, and their interaction P_*Gen* × *CsA*_. Other comparisons were performed either using *t*-test or in **(A–C,E)** Mann–Whitney test.

Given the role of AMPK in mitochondrial biogenesis and mitophagy, as well as the fact that mitochondria represent the main source of ATP to sustain cardiac contractile function, we compared the respiratory parameters of mitochondria isolated from left ventricles of CTR and KO mice at 10 weeks after tamoxifen administration by oxygraphy (Figure 8). With glutamate and malate as substrates that provide electrons mainly to respiratory complex I, KO mice showed a significantly reduced ADP-stimulated respiration as compared to CTR mice (state 3; Figure 8A). No such difference was seen with complex II substrate succinate or palmitoylcarnitine that enters β-oxidation and supplies electrons also further downstream of complex I (Figures 8B,C), or at maximal respiratory capacity with uncoupler (Figure 8D). Next, we analyzed the activity of respiratory complexes and related enzymes individually. Consistent with the divergent respirometry data for complex I substrates, we found reduced enzymatic activity of complex I in hearts from KO mice as compared to CTR (Figure 8E). AMPK KO mice also showed reduced enzymatic activity of complex IV (Figure 8F). In agreement with respirometry data for palmitoylcarnitine being similar for both phenotypes, activity of carnitine:palmitoyltransferase (CPT1 + 2) was unchanged (Figure 8G). In spite of alterations in mitochondrial function observed in KO mice, the abundance of respiratory chain complexes in total heart extracts was unchanged as verified by immunoblotting (data not shown). This suggests differences at the post-translational level. There was neither a detectable effect of KO on calcium retention capacity linked to mitochondrial permeability transition and its sensitivity to cyclosporine A (Figure 8H). Despite inhibition of complex I in KO animals, we neither observed differences of oxidative stress markers in cardiac tissue between CTR and KO mice, as assessed by analysis of TBARS and thiol (−SH) groups (Figures 8I,J).

### Mitochondrial Ultrastructure

**FIGURE 9:**
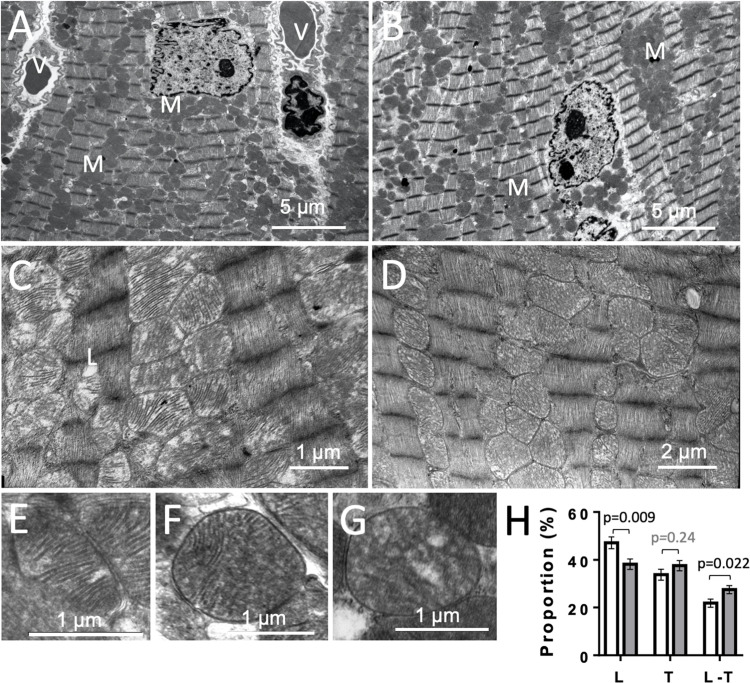
Cardiac ultrastructure in control and AMPKα1α2-KO mice. **(A,B)** Panoramic electron microscopy view of a control **(A)** and AMPKα1α2-KO **(B)** myocardium 10–16 weeks after tamoxifen administration. M, mitochondria; V, vessel. **(C,D)** Pleomorphic mitochondria in control **(C)** and AMPKα1α2-KO **(D)** hearts. **(E–H)** The three basic morphologies of mitochondrial cristae: lamellar **(E)**, mixed lamellar-tubular **(F)** and tubular **(G)** and their prevalence in control and AMPKα1α2-KO myocardium **(H)**. Mitochondrial cristae morphometry was realized on 1291 mitochondria (651 for control and 640 for KO mice) on 46 and 39 micrographs from control KO mice (*n* = 6 control and 3 KO). Each mitochondrion was classified into one of the following morphological categories: tubular T, lamellar L, or mixed (lamellar-tubular L-T); control (white bar), KO (gray bar). The relative percentage of the three basic morphologies was calculated; comparisons were done using *t*-test.

To get further insight into potential mitochondrial alterations, we examined heart ultrastructure with a focus on mitochondria using electron microscopy. There were no obvious differences in cardiac ultrastructure and overall mitochondrial volume between CTR and KO mice (Figures 9A,B). However, mitochondrial ultrastructure revealed differences in cristae morphology, with more prevalent lamellar cristae in CTR hearts and more prevalent mixed cristae (lamellar-tubular) in KO (Figures 9C–H). Given the dynamics of the mitochondrial inner membrane, this could reflect functional differences as those observed above.

## Discussion

In this work we studied a new transgenic animal model, the inducible, cardiomyocyte-specific knockout mouse deficient for both isoforms of the catalytic AMPKα-subunit, α1 and α2. This KO model not only allows to specifically address cardiac functions of AMPK, since it avoids e.g., compensatory upregulation of the minor AMPKα1 isoform in AMPKα2 KO mice [as observed in gastrocnemius muscle of AMPKα2 mice (Viollet et al., 2003)] or potential cross-talk with other organs in whole body knock-out models (Davis et al., 2012). Induction of the knock-out at the adult stage also circumvents inherent caveats of constitutive knock-outs, mainly compensatory mechanisms during embryogenesis and development. While a beneficial role of cardiac AMPK in pathological processes like ischemia-reperfusion has been identified by these earlier AMPK knock-out models and is well described (Viollet et al., 2009; Zaha and Young, 2012; Salt and Hardie, 2017), activation of AMPK in response to physiological stimuli like an increase in cardiac workload is still incompletely understood in terms of threshold, extent, and role for increased ATP supply in response to higher energy demand. In the present study, we address more specifically such AMPK functions in the physiological range. Indeed, we describe AMPK-dependent limitations of cardiac functions under higher workload. We further examine the structural and functional basis of these limitations, and find a deficiency in mitochondrial functions and cell energetics developing over time.

Cre-loxP mediated, cardiomyocyte-targeted deletion of both AMPKα isoforms occurred in a large majority of these muscle cells as seen by the important reduction of AMPKα isoforms in whole heart, to a degree comparable with other studies using this technique in mouse heart (Liu et al., 2015). The KO was specific for cardiomyocytes and remained stable from week 2 after induction onward. We also confirmed that in mouse heart, AMPKα2 is much more abundant than α1 in cardiomyocytes, while the inverse is observed for other cell types.

This AMPK KO model did not reveal a discernable phenotype at baseline as compared to CTR mice. Growth and food intake were comparable, as were cardiomyocyte size and ultrastructure. In particular, heart function in KO mice was unchanged, similar to most other models of AMPK invalidation. In the constitutive, whole-body AMPKα2 KO mice, basal cardiac contractility was unaffected in echography of anesthetized mice (Zarrinpashneh et al., 2006) or in isolated perfused hearts at normoxia (Athea et al., 2007; Carvajal et al., 2007). Few differences in cardiac function at baseline were reported for AMPK invalidation in both heart and skeletal muscle, using overexpression of a dominant-negative kinase-dead AMPKα2 (Russell et al., 2004), and only muscle-specific AMPK β1/β2 deletion showed more pronounced cardiac dysfunction (Sung et al., 2015), possibly due to absent AMPK signaling during development as compared to our model.

An important new finding with our cardiac AMPK KO model are alterations that appear under increased workload and with advanced age, i.e., prolonged KO. Mainly, several functional parameters of the heart were impaired under increased workload such as induced by dobutamine. Cardiac output increased significantly in response to dobutamine in CTR animals, while this was not the case in KO mice. Consistent with such impairments, exercise capacity of KO as compared to CTR animals was limited under the increased workload of endurance exercise. The inability to increase cardiac output under stress conditions as revealed by dobutamine may be causative for the reduced exercise tolerance. Indeed, cardiac function can limit the ability of the cardiorespiratory system to deliver oxygen to exercising muscles (Bassett and Howley, 2000) and is thus an important predictor of endurance performance.

In skeletal muscle, exercise-induced increase of AMPK activity is one of the hallmarks of AMPK signaling (Lee-Young et al., 2009). In heart, the link between increased workload under physiological stimuli like endurance exercise, AMPK activation and cardiac function is far from being clear. On the one hand, exercise-induced and exercise intensity-dependent increase in AMPK activity has been demonstrated in rats (Coven et al., 2003). On the other hand, transgenic mice constitutively expressing a cardiac-specific dominant-negative AMPKα2 (Musi et al., 2005) did not show abnormal exercise capacity. Chronic adaptive changes cannot be ruled out here, and one has to consider that heart has a unique metabolic stability and energy homeostasis, maintained by metabolic networks and a concerted action of several metabolic and signaling kinases, able to manage transitions in energy demand (Saks et al., 2006; Neumann et al., 2007). In contrast, there are reports on lower exercise capacity in animals bearing AMPK deficiency in both skeletal and heart muscles: constitutive whole-body AMPK β2 KO showing downregulation of AMPKα1 and α2 in skeletal muscle and α2 in the heart (Steinberg et al., 2010), and kinase-dead AMPKα2 overexpressing mice, downregulating endogenous AMPK in both muscles (Lee-Young et al., 2009). In both models, authors attributed impaired exercise tolerance to AMPK deficiency in skeletal muscle, not in the heart. Indeed, markedly impaired exercise tolerance can be seen in mice with AMPK deficiency solely in skeletal muscles [AMPKα1α2 double-knockout (Lantier et al., 2014)]. Our results show however that also cardiac AMPK deficiency can impair exercise tolerance. These new data suggest that even effects reported for mixed cardiac and skeletal muscle AMPK deficiency could at least partially be due to limited cardiac function.

Lack of cardiac AMPK could affect cardiac performance and ultimately exercise tolerance in two ways, either acutely by a lack of exercise-induced AMPK signaling, or via chronic alterations leading e.g., to impaired cardiac mitochondria. Both could in the acute phase of endurance exercise lead to reduced energy reserves that limit contractile function. To compare AMPK activation and energy state in KO and CTR animals under identical conditions, we could not use the endurance exercise protocol, but applied instead a standardized exercise of rather moderate intensity, which could be performed by both genotypes. Under these conditions, after exercise, there was only a decreasing trend in energy reserve in both genotypes, and no significant activating AMPK phosphorylation, neither in KO nor CTR animals. Indeed, several studies report a lack of cardiac AMPK activation as a result of the increased workload, e.g., in swine hearts treated with dobutamine (Hall et al., 1996), in isolated rat hearts stimulated with epinephrine (Goodwin and Taegtmeyer, 1999), or in isolated working heart where work was modulated by changing the afterload (Beauloye et al., 2002). It is possible that our moderate exercise protocol did not reach the necessary threshold for detectable AMPK phosphorylation, as the extent of AMPK activation in hearts was shown to be exercise intensity-dependent (Coven et al., 2003).

As mentioned above, AMPK signaling could also be important in the long term to fully establish the mechanisms that maintain metabolic stability in the heart. We therefore looked into different mitochondrial functions. Consistently we found that AMPK deficiency resulted in impaired respiratory function of cardiac mitochondria. KO mice showed reduced respiration with complex I, but not complex II substrates, and had impaired activity of complexes I and IV, associated with a shift in mitochondrial cristae morphology from lamellar to mixed lamellar-tubular, but without apparent difference in mitochondrial density. No KO effects were found for calcium retention capacity and ROS generation, the latter indicated by assessment of TBARS and thiol (−SH) groups. A similar mitochondrial phenotype was reported for AMPK deficiency in skeletal muscle (Lantier et al., 2014), where a marked decrease in exercise capacity was also linked to defects in oxidative phosphorylation, in particular complex I. Also in whole-body AMPKα2 KO mice, a reduced function of respiratory complexes was found and linked to perturbed cardiolipin homeostasis observed in this model (Athea et al., 2007). We conclude that chronic AMPK signaling in the heart is essential for mitochondrial oxidative function, substrate utilization and inner membrane ultrastructure in a way that allows to fulfill energy demand at high workload.

The second new finding with our cardiac AMPK KO model are alterations observed with advanced age. One year after tamoxifen administration, AMPKα deletion induced a decrease in basal metabolic rate (oxygen uptake, energy expenditure) accompanied by a trend to lower spontaneous locomotor activity (P_*Gen*_ = 0.09). Diminished spontaneous activity was also found in mice deficient for LKB1 in skeletal and heart muscles. Knock-out of LKB1, the upstream kinase of AMPK, resulted in decreased voluntary running and reduced mitochondrial marker enzymes in muscle (Thomson et al., 2007). Lower basal metabolic rates were observed earlier in humans bearing mutations in the KSR2 gene which leads to deficient AMPK signaling [(Pearce et al., 2013); reviewed in Hardie (2014)]. Strikingly, in our cardiac AMPK KO model, energy reserves at baseline did not differ at 24 weeks after tamoxifen administration, but showed trends to deterioration in KO mice at 52 weeks (e.g., decreased ATP, *p* = 0.05). Reduction in cardiac glycogen has been previously observed in several models of AMPK deletion (Russell et al., 2004; Carvajal et al., 2007; Sung et al., 2015). We conclude that chronic AMPK signaling in the heart is involved in maintaining its basic metabolic functions with advanced age.

Taken together, our study promotes the view of cardiac AMPK as a continuous regulator of the cardiac metabolic network, among others shaping mitochondrial function for a sustained response to workload transitions and during advanced age. Acute energy stress and resulting strong AMPK activation seems to occur only in extreme situations, such as reported for ischemia/reperfusion (Dyck and Lopaschuk, 2006; Viollet et al., 2009; Zaha and Young, 2012; Salt and Hardie, 2017).

## Data Availability Statement

The original contributions presented in the study are included in the article/Supplementary Material, further inquiries can be directed to the corresponding author/s.

## Ethics Statement

The animal study was reviewed and approved by Animal Ethics Committee of the Université Grenoble Alpes (453#15816, 477#19471) and French Ministry of Higher Education, Research and Innovation (authorization #15816-2018070218298485, #19471-2019022612196859 v3).

## Author Contributions

MT-S, LK, HD, CG, CZ, BV, and US conceived the study and designed the experiments. MT-S, LK, PP, RI, SA, FL, CT, CC-R, AU, IH-F, HD, and CZ carried out the experiments and contributed to sample collection. MT-S, LK, PP, RI, CC-R, FL, and HD analyzed the data. MF and BV generated mouse model. MT-S and US wrote the manuscript. All authors have read, revised and approved the final version of the manuscript.

## Conflict of Interest

The authors declare that the research was conducted in the absence of any commercial or financial relationships that could be construed as a potential conflict of interest.

## Publisher’s Note

All claims expressed in this article are solely those of the authors and do not necessarily represent those of their affiliated organizations, or those of the publisher, the editors and the reviewers. Any product that may be evaluated in this article, or claim that may be made by its manufacturer, is not guaranteed or endorsed by the publisher.
