# Supplementary material for: Role of Cardiac AMP-Activated Protein Kinase in a Non-pathological Setting: Evidence From Cardiomyocyte-Specific, Inducible AMP-Activated Protein Kinase α1α2-Knockout Mice
Source: Front Cell Dev Biol. 2021 Oct 18;9:731015. doi: 10.3389/fcell.2021.731015 (PMC8558539; doi:10.3389/fcell.2021.731015)

Supplementary Material

Supplementary Figures and Tables

Supplementary Table 1. Cardiac function measured by echocardiography before and after KO induction by tamoxifen.

	- 1 w			10 w			52 w			2-way RM Anova		
	C	KO	$p_{KO\ vs\ C}$	C	KO	$p_{KO\ vs\ C}$	C	KO	$p_{KO\ vs\ C}$	P_{Gen}	P_{Time}	$P_{Gen\ x\ Time}$
AoV VTI (mm)	41.88 ± 2.15	41.69 ± 2.00	0.965	35.99 ± 2.82	39.96 ± 4.32	0.359	31.43 ± 1.64	38.33 ± 2.96	0.177	0.326	**0.026**	0.326
LVOT (mm)	1.19 ± 0.02	1.18 ± 0.02	0.647	1.27 ± 0.02	1.27 ± 0.01	0.903	1.30 ± 0.03	1.30 ± 0.02	0.921	0.930	**< 0.001**	0.801
HR t0 (BPM)	450.20 ± 9.00	460.50 ± 9.90		471.55 ± 9.32	480.31 ± 15.36		463.89 ± 16.35	456.77 ± 11.71		0.656	0.194	0.848
HR tend (BPM)	413.55 ± 15.34	412.38 ± 11.20	0.955	465.45 ± 13.36	460.88 ± 19.50	0.826	418.89 ± 15.73	372.77 ± 8.82	0.052	0.250	**< 0.001**	0.194
IVCT (ms)	18.81 ± 2.05	20.57 ± 2.08		15.07 ± 1.46	15.94 ± 1.33		17.61 ± 0.97	20.22 ± 1.82		0.333	0.146	0.928
IVRT (ms)	17.29 ± 0.69	16.73 ± 1.28	0.960	15.12 ± 0.87	16.31 ± 0.99	0.578	18.37 ± 1.22	22.89 ± 1.15	0.009	0.078	**0.004**	0.113
MV A (mm/s)	419.50 ± 34.47	382.40 ± 26.52	0.398	355.18 ± 28.98	345.61 ± 29.81	0.827	328.00 ± 48.78	303.80 ± 24.04	0.347	0.316	**0.031**	0.793
MV E (mm/s)	604.92 ± 47.58	586.82 ± 35.44	0.734	521.10 ± 24.59	522.93 ± 36.70	0.937	483.53 ± 39.96	468.82 ± 38.34	0.694	0.737	**0.003**	0.917
MV E/A	1.48 ± 0.08	1.58 ± 0.08		1.39 ± 0.09	1.60 ± 0.09		1.65 ± 0.17	1.58 ± 0.10		0.229	0.649	0.621
LVPA;d (mm)	0.69 ± 0.04	0.67 ± 0.03	0.573	0.77 ± 0.03	0.74 ± 0.02	0.414	0.81 ± 0.04	0.79 ± 0.02	0.353	0.296	**< 0.001**	0.914
LVPA;s (mm)	1.04 ± 0.05	1.01 ± 0.05	0.652	1.22 ± 0.05	1.16 ± 0.05	0.335	1.18 ± 0.07	1.18 ± 0.03	0.768	0.462	**< 0.001**	0.841
LVID;d (mm)	4.02 ± 0.09	4.05 ± 0.08		3.94 ± 0.07	3.91 ± 0.08		4.12 ± 0.15	4.10 ± 0.08		0.962	0.331	0.919
LVID;s (mm)	2.92 ± 0.10	2.98 ± 0.10	0.656	2.53 ± 0.10	2.63 ± 0.08	0.455	2.95 ± 0.11	2.90 ± 0.08	0.850	0.651	**< 0.001**	0.770
LVPW;d (mm)	0.72 ± 0.02	0.68 ± 0.03		0.84 ± 0.03	0.87 ± 0.03		0.94 ± 0.06	0.92 ± 0.02		0.700	**< 0.001**	0.407
LVPW;s (mm)	1.14 ± 0.05	1.00 ± 0.03	**0.013**	1.24 ± 0.04	1.27 ± 0.04	0.626	1.31 ± 0.05	1.31 ± 0.03	0.948	0.313	**< 0.001**	**0.054**
EF (%)	53.90 ± 1.87	52.22 ± 2.37	0.570	65.96 ± 2.08	61.53 ± 1.94	0.137	54.61 ± 2.38	56.65 ± 1.65	0.633	0.511	**< 0.001**	0.267
FS (%)	27.58 ± 1.19	26.70 ± 1.51	0.663	36.09 ± 1.65	32.80 ± 1.36	0.105	28.15 ± 1.57	29.44 ± 1.13	0.688	0.481	**< 0.001**	0.248
LV Mass (mg)	99.57 ± 2.60	95.41 ± 4.03	0.477	115.60 ± 3.53	113.76 ± 3.37	0.753	137.86 ± 7.01	134.11 ± 4.56	0.427	0.352	**< 0.001**	0.902
LV Mass corr (mg)	79.66 ± 2.08	76.32 ± 3.23	0.639	92.48 ± 2.83	91.01 ± 2.70	0.371	110.29 ± 5.61	107.29 ± 3.65	0.404	0.284	**< 0.001**	0.973
LV/BW (mg/g)	3.28 ± 0.08	3.16 ± 0.10	0.671	2.98 ± 0.10	3.00 ± 0.11	0.435	2.55 ± 0.17	2.53 ± 0.12	0.961	0.644	**< 0.001**	0.788
LV Vol;d (µL)	71.22 ± 3.57	72.52 ± 3.15		68.02 ± 2.92	66.78 ± 3.09		75.84 ± 5.66	74.66 ± 3.23		0.952	0.282	0.921
LV Vol;s (µL)	33.26 ± 2.76	35.36 ± 2.88	0.550	23.56 ± 2.15	25.96 ± 1.95	0.495	34.17 ± 3.04	32.56 ± 2.08	0.764	0.662	**< 0.001**	0.715
AoV SV (µL)	46.46 ± 1.93	45.94 ± 2.26		45.18 ± 3.01	50.82 ± 5.49		40.35 ± 3.43	50.87 ± 3.43		0.189	0.746	0.294
AoV CO (mL/min)	19.08 ± 0.85	18.83 ± 1.05		21.13 ± 1.76	24.00 ± 3.70		16.87 ± 1.46	18.95 ± 1.31		0.518	0.078	0.709

Data are presented as Mean±SEM (N=11 Control, 16 KO) and analysed by 2-way Repeated Measure Anova followed by Holm-Sidac test.
A value of P or p<0.05 (for interaction P<0.1) was considered statistically significant (in bold).
Highlighted in grey, data with significant effect of time.

The detailed description of abbreviations, measurements and calculations:
AoV Flow, ascending aorta view
AoV VTI, Aorta velocity time integral, PW Doppler
LVOT, Left ventricular outflow tract lenght, B-mode
HR, heart rate, ECG
MV Flow, 4-chambers view, mitral valve
IVCT, Isovolumic contraction time, PW Doppler
IVRT, Isovolumic relaxation time, PW Doppler
MV A, Mitral valve A (atrial) velocity, PW Doppler
MV E, Mitral valve E (early) velocity, PW Doppler
Calculation
MV E/A, Mitral valve E/A ratio, MV E / MV A
SAX M-Mode, small axe view
LVAW;d, Inter-ventricular anterior wall (diastole), M-Mode
LVAW;s, Inter-ventricular anterior wall (systole), M-Mode
LVID;d, Left ventricular internal diameter (diastole), M-Mode
LVID;s, Left ventricular internal diameter (systole), M-Mode
LVPW;d, Left ventricular posterior wall (diastole), M-Mode
LVPW;s, Left ventricular posterior wall (systole), M-Mode
Calculation
EF, LV ejection fraction, 100*((LV Vol;d-LV Vol;s)/LV Vol;d)
FS, LV fractional shortening, 100*((LVID;d-LVID;s)/LVID;d)
LV Mass (mg), LV mass uncorrected, 1.053*((LVID;d+LVPW;d+IVS;d)^3 - LVID;d^3)
LV Mass corr (mg), LV mass corrected, LV mass * 0.8
LV/BW (mg/g), LV mass corrected for body weight, LV mass corrected/BW
LV Vol;d, Left Ventricle volume diastole, ((7.0/(2.4+LVID;d))*LVID;d^3
LV Vol;s, Left Ventricle volume systole, ((7.0/(2.4+LVID;s))*LVID;s^3
Ao SV, Stroke volume, (π/4)* LVOT^2*AoV VTI
Ao CO, Cardiac output, (AoV SV*HR)/1000

Supplementary Table 2. Weight of liver, kidneys and spleen 52 weeks after KO induction by tamoxifen.

	C	KO	$P_{KO\ vs\ C}$
Body weight (g)	43.16 ± 2.86	43.48 ± 1.81	0.920
Liver (g)	1.819 ± 0.137	1.921 ± 0.097	0.541
Kidneys (g)	0.433 ± 0.015	0.461 ± 0.014	0.204
Spleen (g)	0.079 ± 0.003	0.084 ± 0.006	0.466

Data are presented as mean ± SEM (n=8 Control, 10 KO) and analysed by Student's t-test.
A value of $P<0.05$ was considered statistically significant.

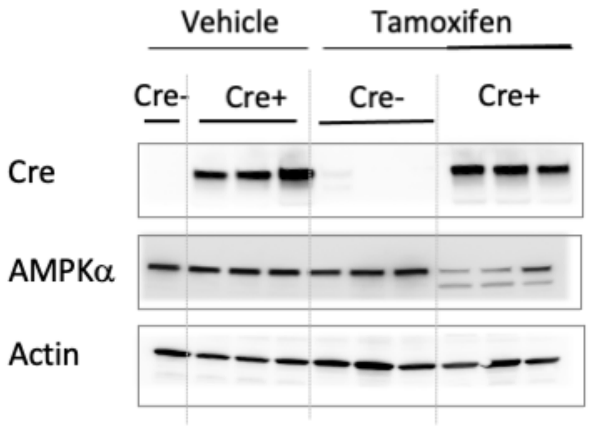

Supplementary Figure 1. Tamoxifen-inducible and Cre-mediated deletion of AMPK α (α1+ α2) analyzed by Western blot in AMPKα1(fl/fl)AMPKα2(fl/fl) (Cre-) and AMPKα1(fl/fl)AMPKα2(fl/fl) (α MHC)-MerCreMer (Cre+) mice 2 weeks after tamoxifen or vehicle administration.

Supplementary Figure 2. Loading controls (Ponceau stainings and tubulin immunoblots) for Figures 2A, 2B and 2D.

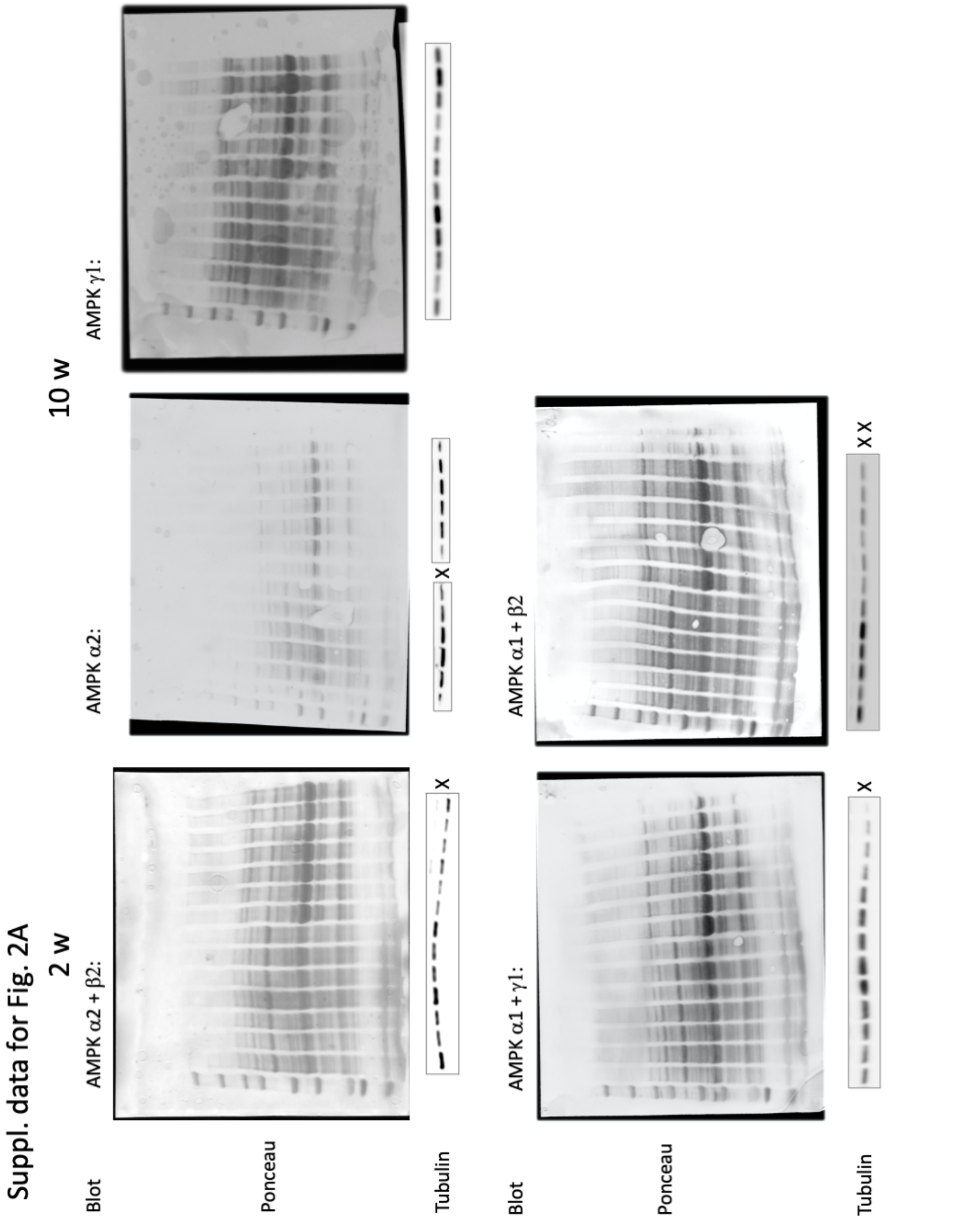

Suppl. data for Fig. 2A

52 w

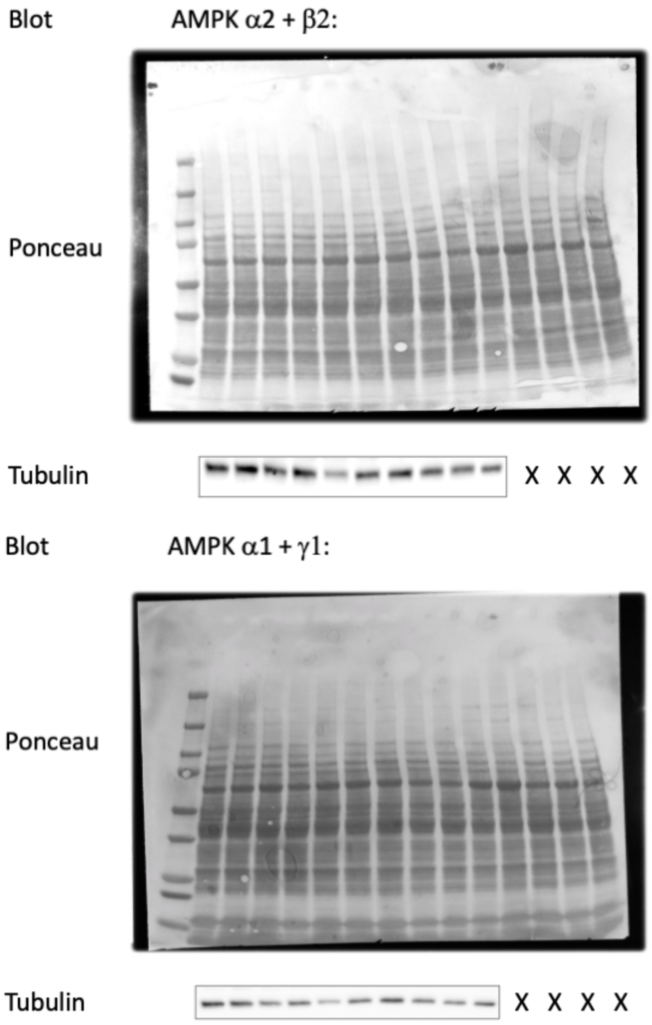

Suppl. data for Fig. 2B

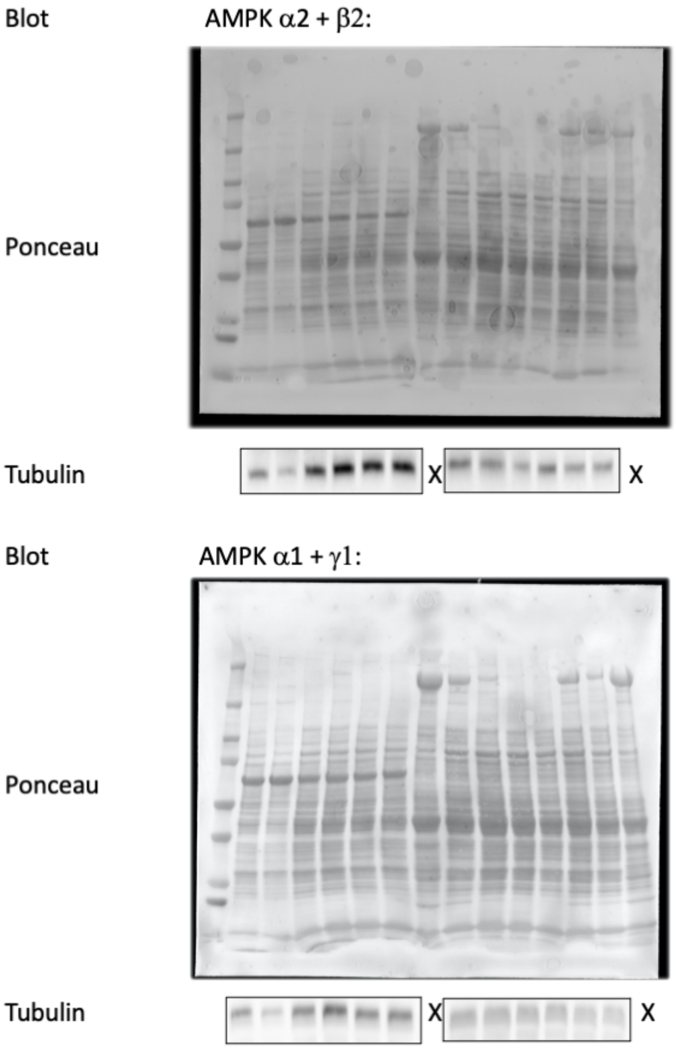

Suppl. data for Fig. 2D

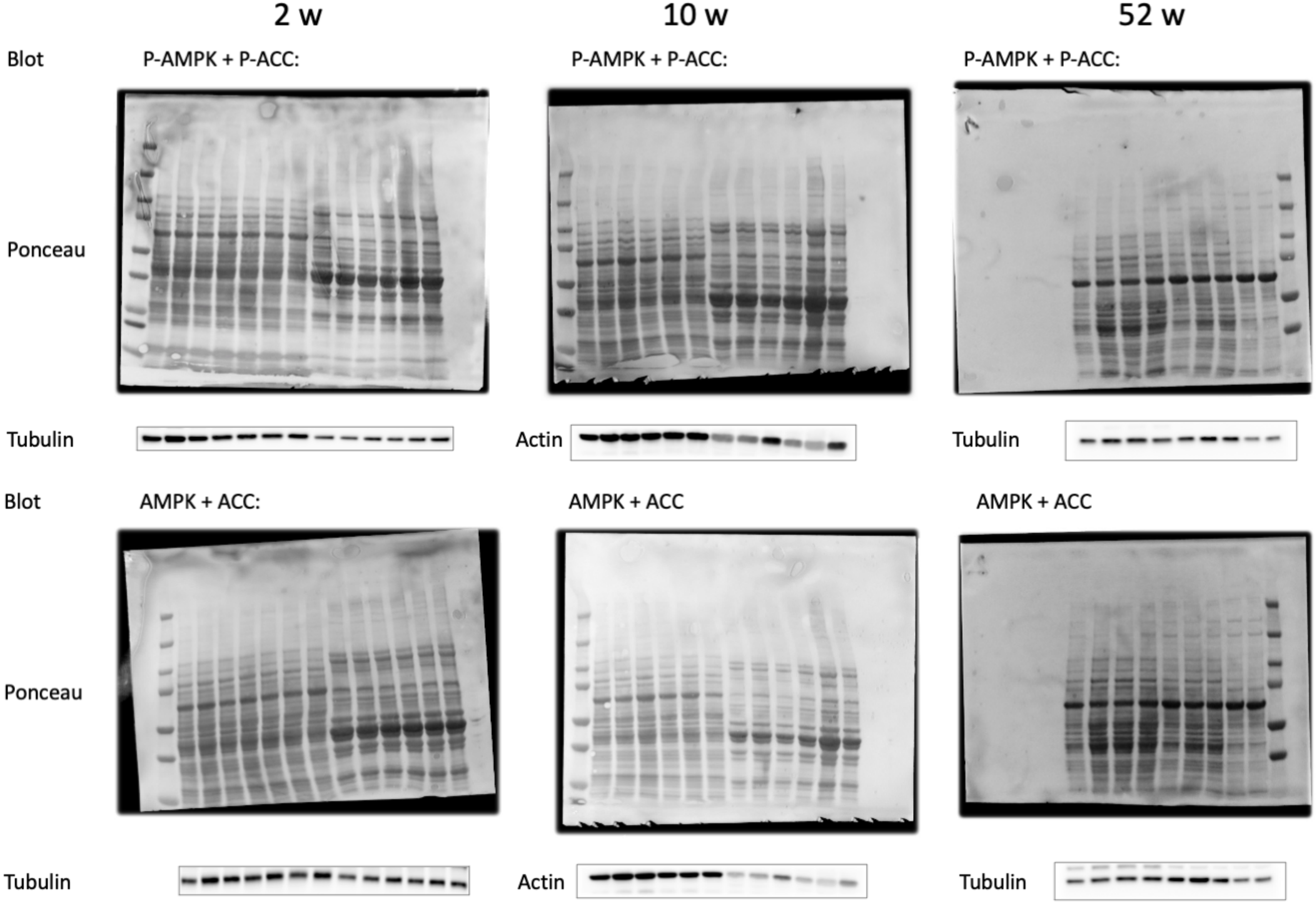

Supplement: Supplementary file 1 [file Data_Sheet_1.pdf]
